# Gordonia bronchialis Bacteremia in a Patient With Burkitt Lymphoma: A Case Report and Literature Review

**DOI:** 10.7759/cureus.30644

**Published:** 2022-10-24

**Authors:** Benjamin J McCormick, Razvan M Chirila

**Affiliations:** 1 Internal Medicine, Mayo Clinic, Jacksonville, USA

**Keywords:** burkitt lymphoma, immunocompromised, bacteremia, gordonia bronchialis, gordonia

## Abstract

*Gordonia* species are gram-positive, partially acid-fast bacteria recognized as pathogens associated with medical devices and catheter-related infections in immunocompetent and immunocompromised hosts. We describe a rare case of *Gordonia bronchialis* bacteremia due to central venous catheter infection in a patient undergoing active chemotherapy for Burkitt lymphoma. We review the diagnosis, treatment, and extent of infections reported throughout medical literature about this rare and emerging pathogen.

## Introduction

The Gordonia (G.) genus of bacteria was first described in 1971 by Tsukamura and belongs to the Actinomycetia class, including Nocardia, Corynebacterium, Mycobacterium, Rhodococcus, and Gordonia genera. Previously classified as Rhodococcus spp., Gordonia spp. infections have been associated with medical procedures and devices, and major pathogens include Gordonia bronchialis, Gordonia sputi, and Gordonia terrae [1]. Speciation of G. bronchialis is achieved via 16S rRNA sequencing and matrix-assisted laser desorption/ionization time-of-flight (MALDI-TOF) mass spectrometry. Other systems are currently ineffective due to database limitations [2,3].

G. bronchialis (formerly Rhodococcus bronchialis) was first genetically sequenced in its entirety in 2010. It is a gram-positive, catalase-positive, partially acid-fast, nitrate-reducing, urease-producing, non-motile, obligate aerobe with a rod-like shape that is known to form into sessile, cord-like communities. It possesses lipoarabinomannan-like lipoglycan like Mycobacterium, which serves as a major virulence factor [4].

G. bronchialis has been reported as a human pathogen in a handful of case reports over the past two decades, which started after an infamous outbreak in 1988 [5]. Seen in both immunocompetent and immunocompromised hosts, G. bronchialis typically presents with site-dependent inflammatory responses with the potential for systemic infection and is associated with significant morbidity. We describe a case of G. bronchialis bacteremia in an immunocompromised host.

## Case presentation

A 56-year-old female with a past medical history significant for diabetes and high-grade B-cell lymphoma undergoing active chemotherapy was admitted for bacteremia and fever. Three months prior, the patient experienced three weeks of painless post-menopausal vaginal bleeding. A CT scan revealed a 7.8 x 6.6 cm cervical/lower uterine segment mass with left iliac chain lymphadenopathy. Biopsy showed high-grade B-cell lymphoma with cytogenetic testing consistent with stage IV Burkitt lymphoma. The patient underwent neoadjuvant chemotherapy with six cycles of R-EPOCH (rituximab, etoposide phosphate, prednisone, vincristine sulfate, cyclophosphamide, and doxorubicin hydrochloride) and 11 intrathecal treatments (methotrexate/cytarabine with hydrocortisone). At the time of presentation to our hospital, she was undergoing cycle six of chemotherapy with daily antimicrobial prophylaxis with fluconazole and valacyclovir.

Six days before admission, she developed fever and diarrhea. Stool Clostridium difficile toxin was negative, blood cultures were obtained, and she was started on empirical levofloxacin. However, she remained intermittently febrile. On the day of admission, blood cultures previously drawn from her tunneled port site grew beaded gram-positive bacilli.

On initial examination, her vital signs were within normal limits. She exhibited chemotherapy-induced alopecia as well as erythema and tenderness at the right sub-clavicular port site. Laboratory findings included the following (reference ranges listed parenthetically): aspartate aminotransferase, 66 U/L (8 to 48 U/L), alanine aminotransferase, 66 U/L (7 to 55 U/L), hemoglobin 9.3 g/dL, (13.2 to 16.6 g/dL), and white blood cell count, 11.8x109/L (3.4 to 9.6 x109/L). Otherwise, the complete blood count and basic metabolic profile were within normal limits. Computed tomography of the chest, abdomen, and pelvis and MRI of the head and neck were negative for acute findings, including no signs of abscess or localized infection.

**Figure 1:**
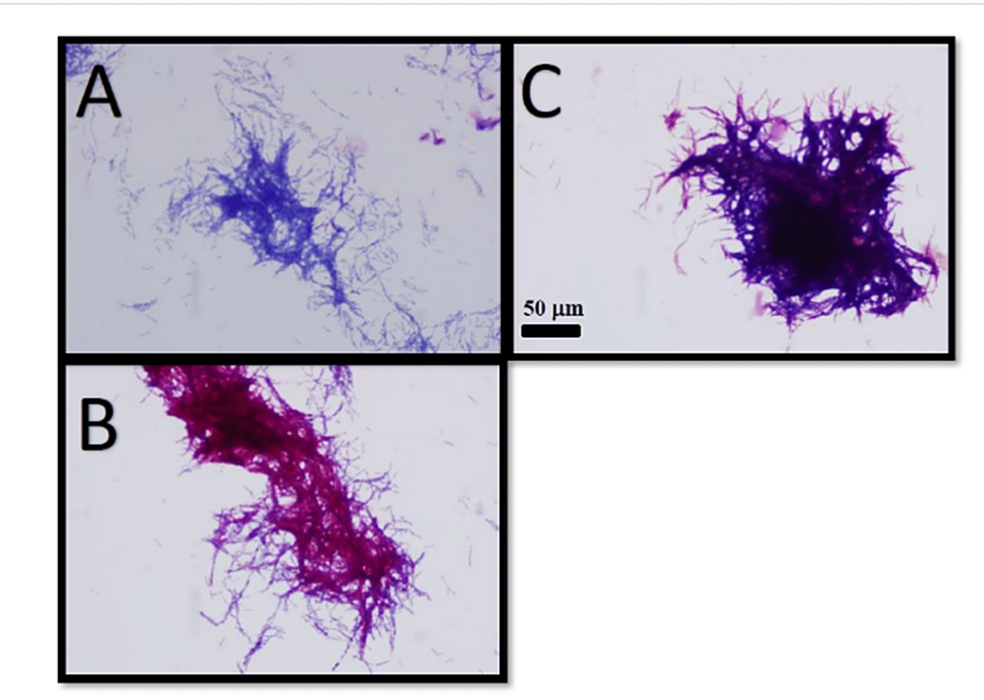
Microbial identification with modified Kinyoun staining Microbial identification testing performed on a positive aerobic culture broth from a serum sample showing gram-positive, partially acid-fast bacilli in cord-like communities via negative Kinyoun stain (A), positive modified Kinyoun stain using sulfuric acid (B), and positive Gram stain (C). Magnification is demarcated.

Gram stain of the cultured port tip revealed beaded gram-positive cocci. Port tip and blood cultures were inoculated and incubated at 37°C on Mycobacteria Growth Indicator Tube (MGIT) liquid culture medium with BD BACTEC MGIT 960 automated monitoring. Port tip and blood cultures were positive for partially acid-fast bacilli. Kinyoun stain and modified Kinyoun stain were performed on positive aerobic culture broths from the catheter tip and serum samples, which demonstrated gram-positive, partially acid-fast bacilli in cord-like communities (Figure 1). The isolate was analyzed via 500-base pair 16S rRNA gene sequencing and MALDI-TOF, which identified Gordonia bronchialis. The indwelling port was removed, and she was empirically started on trimethoprim-sulfamethoxazole and imipenem. All subsequent blood cultures were negative. In vitro isolate susceptibility testing with minimal inhibitory concentrations (MIC in mg/mL) was performed on MGIT broth media via conventional broth microdilution with growth at specific breakpoints indicative of resistance [6]. Intermediate resistance was observed with clarithromycin (MIC 4), doxycycline (MIC 2), and minocycline (MIC 2). The isolate was susceptible to amoxicillin/clavulanate (MIC ≤ 2/1), cefepime (MIC ≤ 1), ceftriaxone (MIC ≤ 4), imipenem (MIC ≤ 2), ciprofloxacin (MIC ≤ 0.12), moxifloxacin (MIC ≤ 0.25), amikacin (MIC ≤ 1), tobramycin (MIC ≤ 1), linezolid (MIC ≤ 1), and trimethoprim-sulfamethoxazole (MIC ≤ 0.25/4.75). Following port removal and negative blood cultures, imipenem-cilastatin 500-500 mg IV q6h was continued for completion of a 28-day course via a peripherally inserted central catheter. Following antibiotic completion, the patient received a robotic total abdominal hysterectomy with bilateral salpingo-oophorectomy (TAH-BSO). Subsequent CT abdomen/pelvis one month after TAH-BSO showed no evidence of genitourinary disease or lymphadenopathy, consistent with remission.

## Discussion

G. bronchialis has been reported as a human pathogen in a handful of case reports over the past two decades, which started after an infamous outbreak in 1988. At that time, seven cases of post-coronary artery bypass graft (CABG) sternal osteomyelitis occurred due to contamination within an operating room [5]. Since the outbreak in 1988, roughly 20 other cases have been reported throughout the medical literature, including reports of sternal infections following CABG, peritonitis following peritoneal dialysis, subcutaneous abscesses following dermal injections and acupuncture, and endophthalmitis following cataract surgery, each of which involved only localized infections without system involvement [7-14]. However, there have been five cases of G. bronchialis bacteremia, which are summarized in Table 1 [2,15-18].

**Table 1:** Summary of reported cases of Gordonia bronchialis bacteremia Abbreviations: CVC, central venous catheter; IV, intravenous; PO, oral; TMP-SMX, trimethoprim-sulfamethoxazole

Year Published	Clinical Manifestations	Age (Years)	Comorbidities/ Exposures	Antibiotic Regimen	Citation
2004	Loculated pleural effusion	58	Diabetes mellitus, sequestrated lung	IV vancomycin & ceftriaxone, then PO amoxicillin-clavulanate	[15]
2007	Ventriculitis	< 1 (45 days)	Premature neonate, intraventricular shunt, central venous catheter	IV amikacin & meropenem	[2]
2011	Pleural effusion	52	Lymphoma, breast cancer, splenectomy, indwelling pleural catheter	IV vancomycin & ceftazidime, then IV TMP-SMX & imipenem-cilastatin, then PO TMP-SMX	[16]
2013	Encephalitis	67	Concurrent HSV encephalitis, diabetes mellitus with HHS	IV cefepime, vancomycin, piperacillin-tazobactam, cefazolin	[17]
2014	Endocarditis	92	Pacemaker placement	IV piperacillin-tazobactam & daptomycin, then IV amoxicillin	[(18]
2022	Indwelling CVC infection	56	Lymphoma with indwelling CVC, diabetes mellitus	IV TMP-SMX & imipenem, then IV imipenem-cilastatin	Current case

Excluding the previously described case of the patient with G. bronchialis bacteremia and concomitant herpes encephalitis without indwelling lines or catheters, all reported cases involve nosocomial etiologies of bacterial invasion, whether via central venous catheter, dermatologic injection, or surgical-site contamination.

There are multiple techniques implemented for laboratory diagnosis of G. bronchialis. Gram stain will show beaded gram-positive bacteria. Kinyoun stain can identify acid-fast bacteria without heat requirements, unlike classical Ziehl-Neelsen staining; however, it is typically negative in Gordonia spp. due to their partially acid-fast nature. To identify Gordonia spp., Kinyoun stain can be modified as a weak acid-fast stain using sulfuric acid instead of hydrochloric acid, enabling the identification of organisms unable to maintain carbol-fuchsin after decolorization with hydrochloric acid [19].

When treating Gordonia spp., initial treatment may consist of a carbapenem or fluoroquinolone with or without an aminoglycoside [1]. A wide variety of antibiotic therapies have shown effectiveness against G. bronchialis, including beta-lactams, carbapenems, tetracyclines, fluoroquinolones, and sulfonamides [2]. Given that only five prior cases of G. bronchialis bacteremia have been reported, no guidelines exist for the duration of antibiotic therapy for catheter-related G. bronchialis bacteremia. Prolonged courses are often implemented, although this is based on extrapolation from treatment guidelines for other partially acid-fast bacilli (e.g., Nocardiosis) rather than culture-guided data.

## Conclusions

Based on these prior case reports and the new case presented, it appears that G. bronchialis is a rare pathogen affecting both immunocompetent and immunocompromised hosts of both adult and pediatric populations, with only about 25 documented cases since 1991. We present the first case of G. bronchialis bacteremia without an associated deep focus of infection (e.g., endocarditis, ventriculitis). It is important to note the predominate nosocomial transmission of this pathogen. G. bronchialis is known to be non-motile with sessile, cord-like community formation; however, there is little data from which one could devise evidence-based guidelines regarding antibiotic choice and whether to remove indwelling lines versus the implementation of antibiotic catheter lock therapy. Accordingly, treatment should be individualized and based on culture isolate antimicrobial susceptibilities combined with close monitoring for clinical response.
